# Accurate Simulation of MPPT Methods Performance When Applied to Commercial Photovoltaic Panels

**DOI:** 10.1155/2015/914212

**Published:** 2015-03-22

**Authors:** Javier Cubas, Santiago Pindado, Ángel Sanz-Andrés

**Affiliations:** ETSI Aeronáuticos, Instituto Universitario de Microgravedad “Ignacio Da Riva” (IDR/UPM), Universidad Politécnica de Madrid, Plaza del Cardenal Cisneros 3, 28040 Madrid, Spain

## Abstract

A new, simple, and quick-calculation methodology to obtain a solar panel model, based on the manufacturers' datasheet, to perform MPPT simulations, is described. The method takes into account variations on the ambient conditions (sun irradiation and solar cells temperature) and allows fast MPPT methods comparison or their performance prediction when applied to a particular solar panel. The feasibility of the described methodology is checked with four different MPPT methods applied to a commercial solar panel, within a day, and under realistic ambient conditions.

## 1. Introduction

Oil prices soared in the second half of the 20th century, the main causes being sociopolitical instabilities such as revolutions or wars, shortage of supply, stock market speculation, or the increasing demand from emerging nations [1]. The economic problems derived from the unstable situation of the energy market forced both the development of renewable energy sources (photovoltaic energy, solar thermal energy, and wind energy) [2–4] and the increase in efficiency in terms of energy generation, transportation, and consumption. In addition, the demand for more efficient energy supply systems has been increased as societies all over the world are increasingly aware of the consequences of the global climate change produced by greenhouse gas emissions.

Among the different renewable energy sources that have been developed and spread in the past half century, the photovoltaic energy is a good example of the above two tendencies. On the one hand, the installed power has increased enormously in some parts of the world, as it has been mainly supported by government investment [5, 6]. On the other hand, technology improvements have produced more efficient solar cells in terms of energy (from 11% efficiency of silicon solar cells in the 1950s [7], so that now it is possible to get gallium-arsenide solar cells of above 30% efficiency [8–11]), cheaper cells based on organic technologies [12–14], and better control processes to maximize the energy supply in every irradiation and temperature conditions.

It is well known that photovoltaic systems are affected by factors that reduce their efficiency such as

- changes on irradiation,
- changes on cells temperature,
- impedance variations at the system output,
- partial shading on the photovoltaic panel.

All these factors change the behavior of the panel, which is normally defined by the output current-output voltage curve (hereinafter, the* I*-*V* curve); see Figure 2 further in the text, and, consequently, the maximum power point (MPP) of the panel is also modified. Therefore, if a photovoltaic system needs to be optimized in terms of power production, implementation on the system of a methodology to “follow” the changes of the MPP is required.

**Figure 1:**
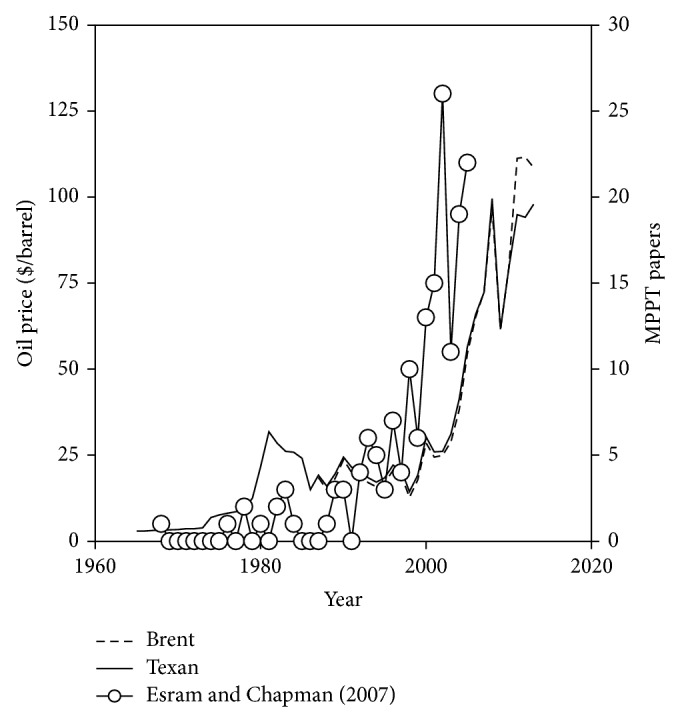
Left axis: oil prices per barrel (Brent and West Texas Intermediate). Right axis: number of papers published between 1968 and August 2005 according to Esram and Chapman.

To the authors' knowledge, the first works to analyze and track the maximum power point of photovoltaic systems were carried out in the late 1960s [15]. Since then, the number of papers produced has broadly increased in line with the oil prices evolution. See in Figure 1 the number of papers related to photovoltaic maximum power point tracking (MPPT) methods that, according to Esram and Chapman, were published between 1968 and 2005, together with the oil prices per barrel (Brent and West Texas Intermediate). In addition, it can be noted that the number of papers per year regarding MPPT in photovoltaic systems has been continually rising as, taking Google Scholar as reference, it has increased from 252 in 2006 to 3220 in 2013. These figures point out the present relevance of improving the MPPT methods.

Papers regarding MPPT methods are not only numerous but diverse, an interesting classification of them being shown by Reza Reisi et al. [16]. These authors divide the different MPPT methods into three categories as follows:

- offline methods, which are those that require information regarding the panel* I*-*V* curve, and also temperature and irradiation levels; among these methods it is possible to find
  - open-circuit voltage and short-circuit current methods, which define the MPP, respectively, as a fraction of the panel open-circuit voltage, *V*_oc_, or the short-circuit current, *I*_sc_;
  - artificial intelligence methods, such as those based on neural networks or fuzzy logic;
- online methods, which are those that require instantaneous measurements of the photovoltaic panel output current and output voltage; the main advantage of these methods is that no information on the panel* I*-*V* curve or regarding irradiation or temperature levels is required; some examples of these methods could be
  - perturbation and observation methods;
  - incremental conductance methods;
  - power peak seeking methods;
  - incremental conductance method;
  - ripple correlation control methods;
- hybrid methods, which are those that combine two different methods, each one from one of the above categories; the offline method is used to get a quick approximation to the MPP whereas the online method is used to improve the result.

Nevertheless, it should also be pointed out that this classification does not include all possible methods. As an example, the power curve fitting method described by Salas et al. and other authors [18, 19] requires an accurate knowledge of both the panel behavior (i.e., the* I*-*V* curve) and the current and voltage measurements at several working points. A more extensive description and classification of MPPT methods can be found at [15, 19–22].

As expected, the high scientific production regarding MPPT methods makes periodical benchmarking necessary, in order to be able to select the best method for every photovoltaic system. Since the 1990s several works aiming at comparing the different MPPT methods can be found at the available literature [15, 16, 18–28]. However, the conclusions from those works should not be widely accepted, as the possible combinations of external factors like the irradiance and temperature variation speed with internal factors such as the system design are an unreliable figure. On the other hand, the specific use/purpose of the generated photovoltaic energy should be taken into account when choosing among the different possible MPPT methods for the considered power system. In line with this statement, one can distinguish between the massive energy supply from solar parks (grid-connected systems) and the supply for small autonomous (stand-alone) systems such as satellites and other spacecraft (although some of them can hardly be considered as small, that is, the International Space Station). In this sense, the differences of MPPT methods applied to mass energy generation or grid-connected systems and to power generation on spacecraft can be underlined. The grid-connected photovoltaic systems need to maximize the production at the lower cost impact, taking into account the fact that a small increase in efficiency can be translated into a huge growth of revenues after some years of operation. This is achieved through MPPT methods with high dynamic and tracking capacities. On the other hand, it is also true that spacecraft require the best possible energy efficiency, but only after considering the reliability of the system [29] and some other associated problems, such as the thermal control [30]. Therefore, in stand-alone systems, especially in spacecraft, simple but more reliable MPPT methods are normally chosen, thereby excluding more efficient, but also more complicated methods.

Validation or comparison of MPPT methods is usually carried out either based on experimental testing [31, 32] results or based on computer simulations [25, 33, 34] although sometimes both kinds of procedures are combined [23, 24]. Experimental comparisons of MPPT methods are logically more realistic, but it should be pointed out that simulations have some important advantages in terms of fast results, cost, and versatility, which make them the best option for algorithms development or efficiency estimations.

One of the most significant difficulties of computational analysis lies in the solar panel behavior simulation. The most common way to do it is through equivalent circuit models, as this has proven to be very accurate [35]. Nevertheless, it must be underlined that different sun irradiation and temperature levels on the panel, as is required by any MPPT validation, involve the recalculation of the different parameters which define the aforementioned equivalent circuit. In many cases, some simplifications of the equivalent circuit are considered, taking into account the fact that multiple recalculations are a quite complicated task. An example of these simplifications could be a circuit without series resistor [34], without parallel resistor [23], or without any resistor [25]. However, series and shunt resistances are directly related to the slope of the* I*-*V* curve at open-circuit and short-circuit points, respectively (*dI*/*dV*|_sc_ ≈ −*R*_*s*_^−1^ and *dI*/*dV*|_oc_ ≈ −*R*_sh_^−1^), and not taking them into account modifies the shape of the* I*-*V* curve and, therefore, the position of the MPP. These simplifications make the behavior of the MPPT numerical simulation slightly diverge from the real performance of the photovoltaic system.

In the present paper a simple, direct, and analytical way to define the behavior of a photovoltaic system under any sun irradiation and cells temperature conditions is presented. Based on the proposed method, solar panel models that fit perfectly to manufacturers' datasheet experimental data can be defined. The equivalent circuit model proposed consists of one diode and two resistors (one in series and the other one in parallel), with no further simplification considered.

Through the methodology presented in this work, accurate simulations of MPPT strategies/algorithms can be achieved, bearing in mind different irradiance and solar cell temperature level and also different DC-DC convertors to be connected to the panel. The aforementioned methodology is based on the 5-parameter equivalent circuit model of a photovoltaic device (i.e., solar panel), the parameter-extraction being analytical (described in Section 2). This analytical extraction to fit the equivalent circuit behavior of the* I-V* curves corresponding to solar cell has proven to be accurate in previous works, in which different photovoltaic technologies (monocrystalline/polycrystalline) [36] or materials (Si/GaAs) [35] were analyzed. Also, if the equations are adjusted to reflect the number of cells connected in series and in parallel, this model can be used to analyze multijunction cells, solar panels, or even groups of solar panels [37]. Nevertheless, it should also be mentioned that the low number of the* I-V* curve points used for extrapolating the curve itself is the greater limitation of the analytical methods, as these points need to be very accurately measured (any deviation at those points will be extrapolated along the* I-V* curve). On the other hand, some optimization of the analytical results can be introduced if some extra data is available [37].

The text is organized as follows. In Section 2 solar panel analytical modeling is described. In Section 3 the way to take into account ambient condition variations in the modeling is shown. In Section 4 four different MPPT methods are presented, whereas calculations and results regarding the MPPT methods comparison carried out with the proposed methodology are included in Section 5. Finally, a case study is included in Section 6, whereas the conclusions of the present work are summarized in Section 7.

## 2. Solar Panel Modeling

**Figure 2:**
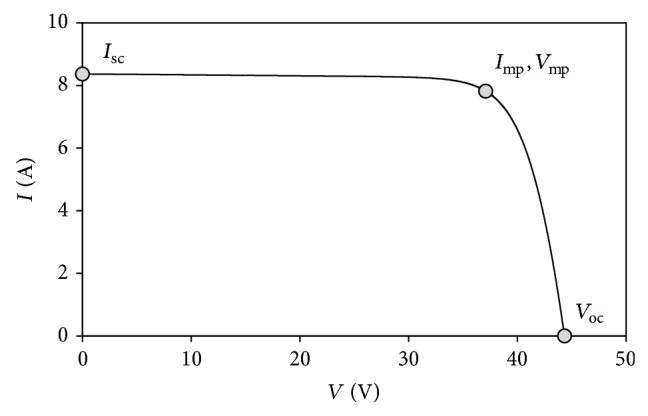
*I*-*V* curve of a solar panel. The three characteristic points (short-circuit, maximum power, and open-circuit points) are indicated on the curve.

**Figure 3:**
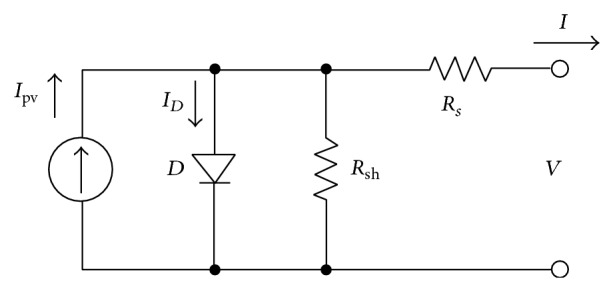
Typical 1-diode equivalent circuit of a solar panel.

In Figure 2, a typical solar cell/panel* I-V* curve is shown. The characteristic points, short-circuit [*I*_sc_, 0], open-circuit [0, *V*_oc_], and maximum power [*I*_mp_, *V*_mp_] points are indicated in the graph. This behavior is similar to the one from a circuit formed by a source of current, *I*_pv_, in parallel to a diode (see Figure 3). Two resistors, one in parallel (shunt resistor), *R*_sh_, and the other one in series (series resistor), *R*_*s*_, are added to take into account losses, such as the ones produced in cell solder bonds, interconnection, and junction box, together with current leakage through the high conductivity shunts across the p-n junction [35].

The equation that defines the behavior of the 1-diode/2-resistors equivalent circuit of Figure 3 is

(1) $$\begin{array}{c} I=I_{\text{pv}}-I_{0}\left[\exp\left(\frac{V+IR_{s}}{aV_{T}}\right)-1\right]-\frac{V+IR_{s}}{R_{\text{sh}}}, \end{array}$$

where, together with the above explained variables (*I*_pv_, *R*_sh_, and *R*_*s*_), *I*_0_ and *a* are, respectively, the reverse saturation current and the ideality factor that takes into account the deviation of the diodes from the Shockley diffusion theory. In accordance with Jain and Kapoor (2005), several theories have been developed to explain the effects that are taken into account by the ideality factor. It is generally accepted that *a* value from *a* = 1 and *a* = 2 takes into account recombination effects inside the cell, whereas larger values take into account shunt resistance effects and nonuniformities on the aforementioned recombination [38]. *V*_*T*_ is not an unknown parameter; it is the thermal voltage of the diode and depends on the charge of the electron, *q*; the Boltzmann constant, *k*; the number of cells in series, *n*; and the temperature, *T*:

(2) $$\begin{array}{c} V_{T}=n\frac{kT}{q}. \end{array}$$

The above expression (1) leaves 5 parameters to be adjusted. This parameter identification is normally performed by fitting the equivalent circuit performance to an* I*-*V* curve obtained for certain working conditions (sun irradiance and cells temperature). However, on many occasions the aformentioned* I*-*V* curve is not available, only information from the manufacturer's datasheet being accesible. In this case, analytical models are probably the best way to approach the equivalent circuit parameter-extraction problem [39–41]. Making use of the characteristic points of the* I*-*V* curve (see Figure 2), the following expressions can be derived from (1) [35]:

(i) short-circuit equation:
  (3) $$\begin{array}{c} I_{\text{sc}}=I_{\text{pv}}-I_{0}\left[\exp\left(\frac{I_{\text{sc}}R_{s}}{aV_{T}}\right)-1\right]-\frac{I_{\text{sc}}R_{s}}{R_{\text{sh}}}, \end{array}$$
(ii) open-circuit equation:
  (4) $$\begin{array}{c} 0=I_{\text{pv}}-I_{0}\left[\exp\left(\frac{V_{\text{oc}}}{aV_{T}}\right)-1\right]-\frac{V_{\text{oc}}}{R_{\text{sh}}}, \end{array}$$
(iii) maximum power point equation:
  (5) $$\begin{array}{c} I_{\text{mp}}=I_{\text{pv}}-I_{0}\left[\exp\left(\frac{V_{\text{mp}}+I_{\text{mp}}R_{s}}{aV_{T}}\right)-1\right]-\frac{V_{\text{mp}}+I_{\text{mp}}R_{s}}{R_{\text{sh}}}, \end{array}$$
(iv) zero derivative for the power at maximum power point equation:
  (6) −ImpVmp=−I0aVT1−ImpVmpRsexp⁡Vmp+ImpRsaVT −1Rsh1−ImpVmpRs.

As four equations are defined for five parameters to be identified, some initial estimation with regard to any of those parameters or to any particular characteristic of the* I*-*V* curve is required. In the first case, calculations are generally started with an estimation of the ideality factor, *a* (which normally lies in the bracket [1,1.5] [39, 42]), as the error of using this starting estimation is reduced (which only affects the curvature of the curve around the maximum power point). Besides, some works in the open literature [43] give information to make such estimations depending on the solar cell technology (Si, Ga-AS, etc.), and, more importantly, this ideality factor can be used as an iteration parameter to obtain an improved approximation to the equivalent circuit [37]. In the second case, some authors use the slope of the* I*-*V* curve at short-circuit or open-circuit points (measured or estimated) to obtain additional boundary conditions equations [35, 41]. Therefore, once the ideality factor *a* has been estimated, the following expressions for the equivalent circuit parameters can be obtained, from (3), (4), (5), and (6), by using the Lambert *W*-function, *W*(*z*), where *z* = *W*(*z*)*e* ^*W*(*z*)^ [36]:

(7) Rs=AW−1BeC−D+C,A=aVTImp,B=−Vmp2Imp−IscVmpIsc+VocImp−Isc,C=−2Vmp−VocaVT+VmpIsc−VocImpVmpIsc+VocImp−Isc,D=Vmp−VocaVT,Rsh=Vmp−ImpRsVmp−RsIsc−Imp−aVTVmp−ImpRsIsc−Imp−aVTImp,I0=Rsh+RsIsc−VocRshexp⁡Voc/aVT,Ipv=Rsh+RsRshIsc.

The equivalent circuit obtained with the above equations can reproduce the considered* I*-*V* curve. However, this curve changes as a function of the sun irradiation and the solar cells temperature, as previously stated. An increase of the temperature produces a higher short-circuit current, *I*_sc_, lower values of the open-circuit voltage, *V*_oc_, and the maximum available power, *P*_mp_ = *I*_mp_ *V*_mp_. These variations of the characteristic points are approximately linear in relation to the temperature, *T*:

(8) Voc,T=Voc,Tr1+βVocT−Tr100,Vmp,T=Vmp,Tr1+βVmpT−Tr100,llIsc,T=Isc,Tr1+αIscT−Tr100,lImp,T=Imp,Tr100+γT−Tr100+βVmpT−Tr,

where *T*_*r*_ is the reference temperature; *βV*_oc_ and *βV*_mp_ are, respectively, the percentage variation of the open-circuit and maximum power point voltages when the temperature increases one degree; *αI*_sc_ and *αI*_mp_ are the percentage variation of the short-circuit and maximum power point currents when the temperature increases one degree; finally, *γ* is the percentage variation of the maximum power with temperature.

As aforementioned, sun irradiation variations also modify the* I*-*V* curve. However, manufacturers normally do not include any information regarding these variations in the solar panel datasheets. Commonly, the shape of the* I-V* curve is considered essentially invariant with irradiation levels within ranges around one solar constant, so this leads to the following equation, considering, respectively, linear and exponential variations of the short-circuit current, *I*_sc_, and the open-circuit voltage, *V*_oc_, with temperature, whereas *R*_*s*_ remains unaffected for temperature variations [44]. Those conditions lead to the following equation [39]:

(9) $$\begin{array}{c} I_{\text{pv},G}=I_{\text{pv},G_{r}}\frac{G}{G_{r}}. \end{array}$$

In the above equation *G* is the irradiance on the cell/solar panel, *I*_pv,*G*_ is the photocurrent delivered by the current source of the equivalent circuit, and *G*_*r*_ and *I*_pv,*G*_*r*__ are the reference values.

Taking all the above statements into account, a simple but accurate (i.e., strictly respecting the data from the manufacturer's datasheet) way to model a solar panel for any irradiation and temperature levels could be summarized as follows.

- Estimate the ideality factor *a*.
- Establish the temperature range in which the solar panel behavior should be modeled and calculate the* I*-*V* curve characteristic points in that range by making use of (8).
- Obtain from expressions (7) the equivalent circuit parameters within the selected temperature range.
- Fit a polynomial expression to the variations of the equivalent circuit parameters as a function of temperature.
- Introduce the effect of the irradiance in the parameter *I*_pv_ by using expression (9).

The above explained process has been applied to the YL280C-30b solar panel manufactured by Yingli Solar (Baoding, China), which will be used in the MPPT simulations included in Section 5. Data regarding the characteristic points of the* I*-*V* curve from the manufacturer's datasheet are included in Table 1. The temperature range considered for the simulations is from 10°C to 65°C.

**Figure 4:**
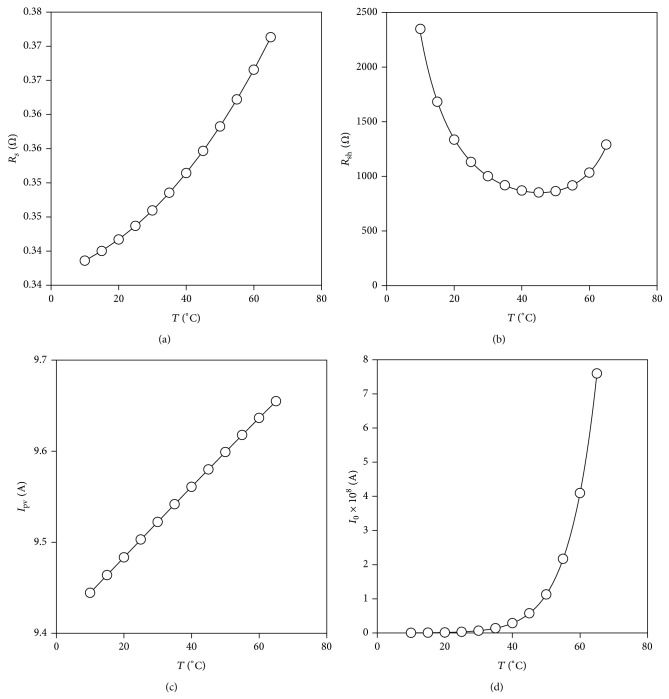
Calculated values of the equivalent circuit parameters *R*_*s*_, *R*_sh_, *I*_pv_, and *I*_0_, for YL280C-30b monocrystalline panel as a function of the temperature, *T*. Calculated points are indicated with symbols whereas the polynomial approximations fitted to those data (10) have been included in each case as solid lines.

With regard to the ideality factor some additional considerations have been made. Although taking into account the silicon monocrystalline technology a value *a* = 1.2 should be selected [43], a lower value has finally been chosen, *a* = 1.05, as this parameter decreases with temperature and the solar panel is supposed to operate at higher temperatures than the reference one, *T* = 25°C, from the Standard Test Conditions (STC). After choosing the value for the ideality factor, the other parameters have been calculated using (7); see Figure 4. The polynomial fittings to the data have also been included in the graphs of the figure, the irradiance level, *G*, being considered in the case of the photocurrent, *I*_pv_; see the following:

(10) IpvT,G=9.50+3.89·10−3ΔTlllllllllllllllllll−1.09·10−6ΔT2−2.96·10−8ΔT3GGr,llllRsT=3.44·10−1+4.23·10−4ΔTllllllllllllllllll+5.90·10−6ΔT2+1.93·10−8ΔT3,lliRshT=8.83·10−4+2.57·10−5ΔTlllllllllllllllllllll−4.02·10−7ΔT2−7.56·10−9ΔT3−1,lllllI0T=exp⁡−2.19·101+1.56·10−1ΔTlllllllllllllllllllllllll−5.20·10−4ΔT2+1.52·10−6ΔT3.

When evaluating an MPPT method the maximum possible power that could be extracted from the panel *p*_max⁡_(*t*) = *I*_mp_(*t*)*V*_mp_(*t*) has to be calculated in every instant, *t*. Then, the efficiency of the method can be estimated with the following expression [45]:

(11) $$\begin{array}{c} \eta_{\text{MPPT}}=\frac{\int_{0}^{T_{T}}p_{\text{MPPT}}\left(t\right)d\tau }{\int_{0}^{T_{T}}p_{\max}\left(t\right)d\tau }, \end{array}$$

where *p*_MPPT_(*t*) is the instantaneous power obtained from the panel using the selected MPPT method and *T*_*T*_ is the total period of time in which the aforementioned MPPT method is evaluated.

In case of evaluation through experimental procedures, the maximum possible power,  *p*_max⁡_(*t*), cannot be directly obtained from the solar panel, being instead estimated from the sun irradiance and temperature levels combined with the panel efficiency at STC. On the other hand, in case of analytical modeling, voltage sampling can be applied to (1) in order to find the highest value of the product* V*·*I* [39], or the maximum power point variables, *I*_mp_ and *V*_mp_, can be obtained from expressions (5) and (6). As these expressions are implicit and coupled, their resolution needs to be done by using numerical calculation or through simplifications in order to reduce the complexity of the calculations [46]. To the authors' knowledge, no method to uncouple variables *I*_mp_ and *V*_mp_ from (5) and (6) has been proposed in the available literature. Nevertheless, this possibility does indeed exist by making the following changes of variable:

(12) φ=−ImpVmp,θ=Vmp+RsImp.

Then, from (5) and (6), it is possible to obtain uncoupled equations as a function of the above new variables:

(13) 1+φRs+Rsh1+φRsaVTRsh +I0exp⁡Rsφ−1Rs+Rshφ−1lllllllllllllllllll×1+φRs+Rsh1+φRs+Ipv+I0RshaVT=0,

(14) θ=aVTRsφ−1Rs+Rshφ−1 ×1+φRs+Rsh1+φRs+Ipv+I0RshaVT.

The variable *φ* can be calculated solving the implicit (13), and then *θ* can be obtained directly from (14). Then, unmaking the change of variables, *I*_mp_ and *V*_mp_, can be derived after only one implicit equation resolution:

(15) $$\begin{array}{c} I_{\text{mp}}=\frac{\varphi \theta }{R_{s}\varphi -1}, \end{array}$$

(16) $$\begin{array}{c} V_{\text{mp}}=\frac{\theta }{1-R_{s}\varphi }. \end{array}$$

This procedure gives the values of current and voltage at the MPP in a very easy way, avoiding voltage sampling or simplifications that could reduce the accuracy of the results. In order to speed up the numerical resolution of (13) it might be useful to start it with the initial value of *φ*_0_ = −*I*_sc_/*V*_oc_.

## 3. Ambient Conditions

**Figure 5:**
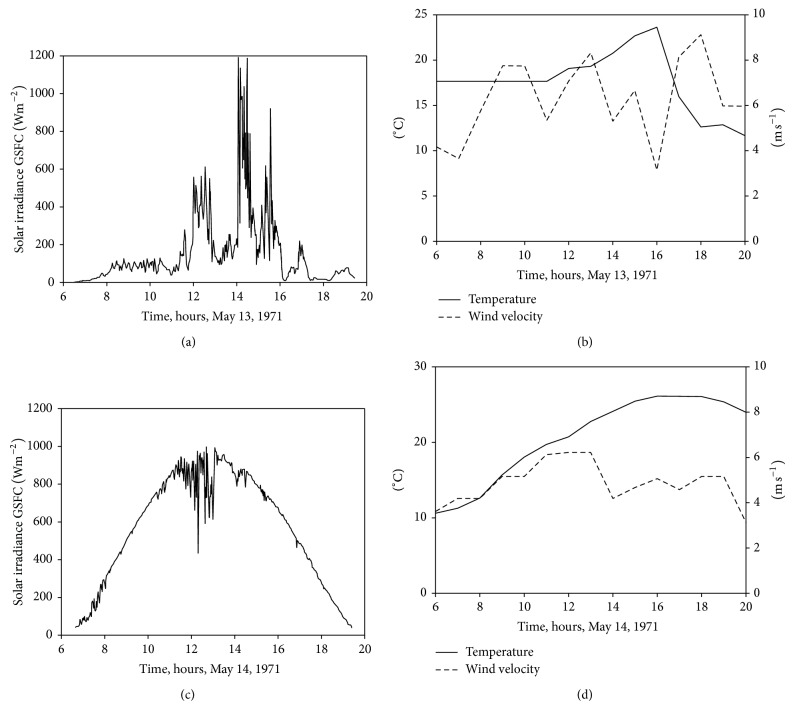
Ambient conditions on May 13, 1971 (a), (b), and May 14, 1971 (c), (d). Solar irradiance is represented in left side, and temperature and wind velocity are in right side.

Two different ambient conditions (sun irradiance and temperature levels) have been considered for the simulations carried out. The first one is a cloudy day, with low but very unstable irradiance level, and the second one is a sunny day; see graphs in Figure 5. These data are, respectively, the irradiance level at the Goddard Space Flight Center (GSFC) on May 13 (cloudy day) and May 14, 1971 (sunny day), and have been extracted from the work by Thekaekara [47]. Temperature and wind speed conditions corresponding to those days have been obtained from the measurements done at Washington, DC, this city being quite close to GSFC [48].

**Figure 6:**
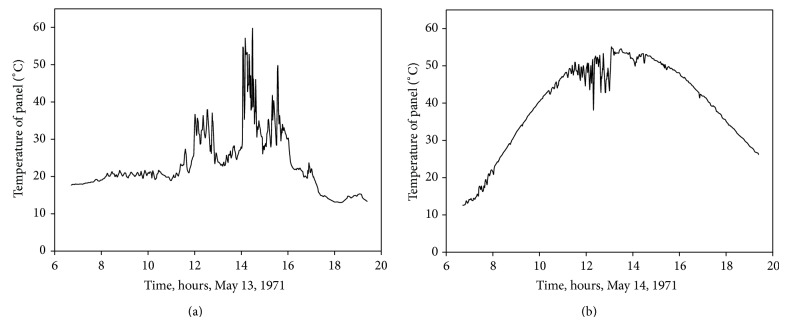
Solar panel temperatures on May 13, 1971 (a), and May 14, 1971 (b), at the Goddard Space Flight Center (GSFC). These graphs were calculated according to ambient conditions for those days; see Figure 5 and (17).

With the data of Figure 5 regarding sun irradiance and ambient temperature it is possible to calculate temperature of the solar panel with the equation

(17) $$\begin{array}{c} T_{p}=T_{a}+\frac{G}{800}\left(\text{NOCT}-20^{{}^{\circ}}\text{C}\right), \end{array}$$

where *T*_*a*_ is the ambient temperature, *G* is the irradiance level, and NOCT is the open-circuit module operation temperature at 800 W/m^2^ irradiance, 20°C ambient temperature, and 1 m s^−1^ wind speed. This last parameter is normally included in the manufacturers' datasheet. Although some authors take wind speed variations into account [49], in the present work they are not considered, as a constant thermal loss coefficient is assumed for wind speeds higher than 1 m s^−1^. Making use of (17), combined with the data from Figure 5, temperature of solar panels located at the Goddard Space Flight Center (GSFC) on March 13 and March 14, 1971, can be estimated; see Figure 6.

**Figure 7:**
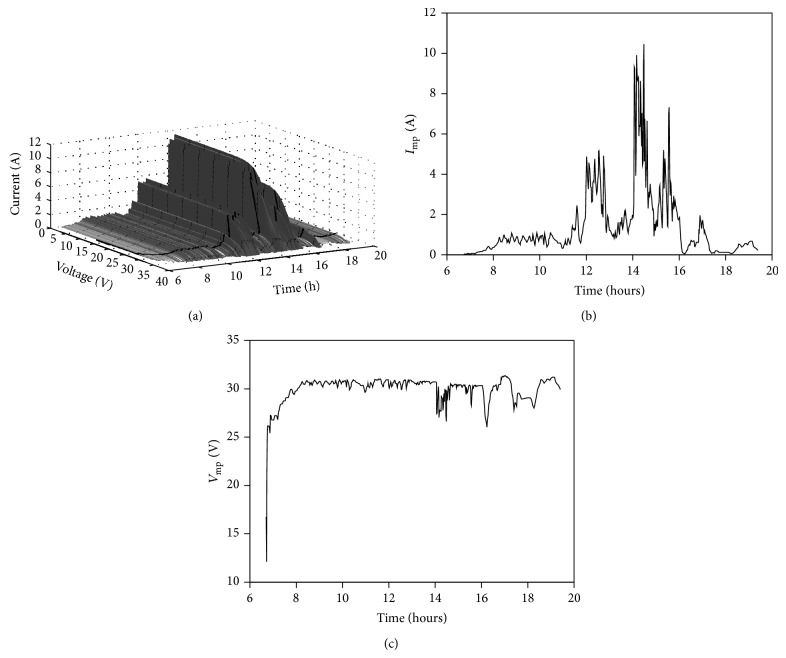
Evolution of the* I*-*V* curve of YL280C-30b monocrystalline solar panel according to ambient conditions, that is, sun irradiance and panel temperature levels, on May 13, 1971 (a), at the Goddard Space Flight Center (GSFC). Evolution of current (b) and voltage (c) at maximum power point (MPP).

**Figure 8:**
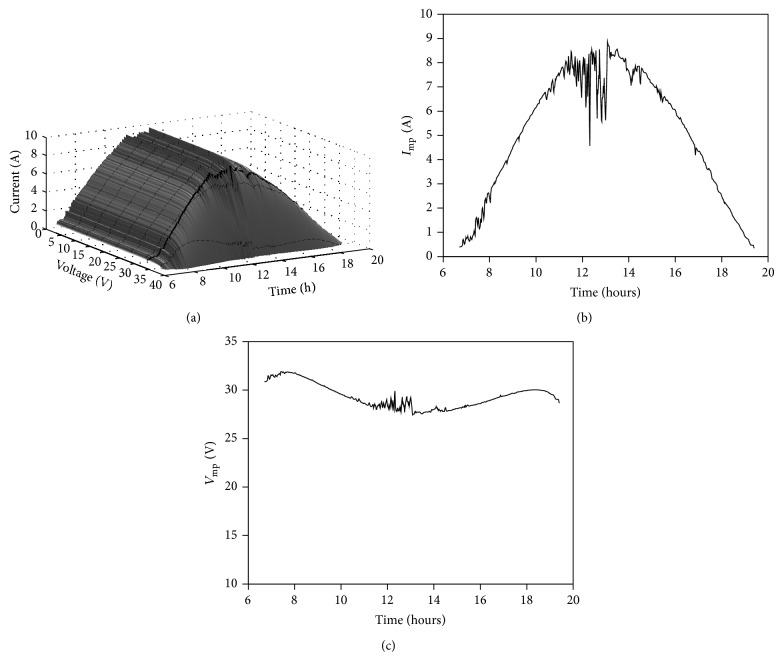
Evolution of the* I*-*V* curve of YL280C-30b monocrystalline solar panel according to ambient conditions, that is, sun irradiance and panel temperature levels, on May 14, 1971 (a), at the Goddard Space Flight Center (GSFC). Evolution of current (b) and voltage (c) at maximum power point (MPP).

Following the procedure described in the previous section and taking into account the aforementioned sun irradiance (Figure 5) and panel temperature (Figure 6) data, it is possible to calculate the* I*-*V* curve of the selected solar panel (YL280C-30b) in each instant of the specified cloudy and sunny days. In Figures 7 and 8, the evolution of the* I*-*V* curve along those days is shown (for one value of time, *t*, the shape of the* I*-*V* curve (see Figure 2) can be appreciated). On the surface plotted in the figures, a thick line has been drawn to indicate the maximum power point (MPP) which, as can be observed, indicates variations regarding the current and voltage at the aforementioned MPP during the chosen days. These current and voltage levels at MPP have been separately plotted in Figures 7(b), 7(c), 8(b), and 8(c). These values of the maximum power available from the solar panel are used in the following section as a reference to compare the studied MPPT methods.

## 4. MPPT Methods Studied

**Figure 9:**
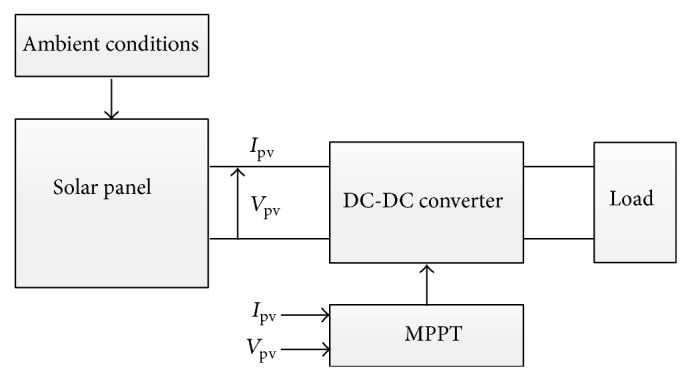
General scheme of a photovoltaic system formed by the solar panels, DC-DC converter controlled by an MPPT method, and load.

As mentioned in Section 1, the aim of the present work is to describe a simple methodology to model a solar panel operating within a wide range of ambient conditions and to analyze different MPPT options for photovoltaic systems (see in Figure 9 the block-diagram corresponding to a photovoltaic system equipped with a MPPT). In the following subsections four MPPT methods are studied as an example of the proposed method:

- constant voltage method,
- open voltage method,
- short-current pulse method,
- perturb and observe method.

**Figure 10:**
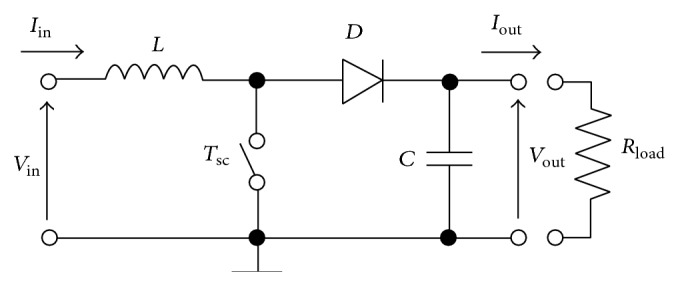
Sketch of the ideal Boost-converter considered.

In this comparison example the ideal Boost-converter has been selected due to its simplicity (see Figure 10). However, it should be underlined that other more sophisticated DC-DC converter possibilities could also be included in the simulation with the proposed methodology, this possibility being out of the scope of the present work, which is focused on the solar panel accurate modeling for MPPT analysis.

The principle of the Boost-converter consists of two operational modes, depending on the position of the switch, *T*_sc_. When the switch is closed (on-state) the inductor *L* stores the energy of the source while the capacitor feeds the load. Otherwise, when the switch is opened (off-state) the only path available for the current is through the diode *D* and the source feeds the load and charges the capacitor. If the converter works with a commutation period, *T*_0_, the duty cycle, *α*, is the fraction of that period in which *T*_sc_ is connected; therefore *α* ranges between 0 (*T*_sc_ is never on) and 1 (*T*_sc_ is always on). For an ideal Boost-converter, the relationship between the input and output variables (current and voltage) is defined by

(18) $$\begin{array}{c} V_{\text{out}}=\frac{V_{\text{in}}}{1-\alpha }, \end{array}$$

(19) $$\begin{array}{c} I_{\text{out}}=\left(1-\alpha \right)I_{\text{in}}. \end{array}$$

Therefore, bearing in mind (17) and (18), the effect of the Boost-converter can be considered as an equivalent resistor, whose value depends on the duty cycle:

(20) $$\begin{array}{c} R_{\text{eq}}=\frac{V_{\text{in}}}{I_{\text{in}}}={\left(1-\alpha \right)}^{2}R_{\text{load}}. \end{array}$$

**Figure 11:**
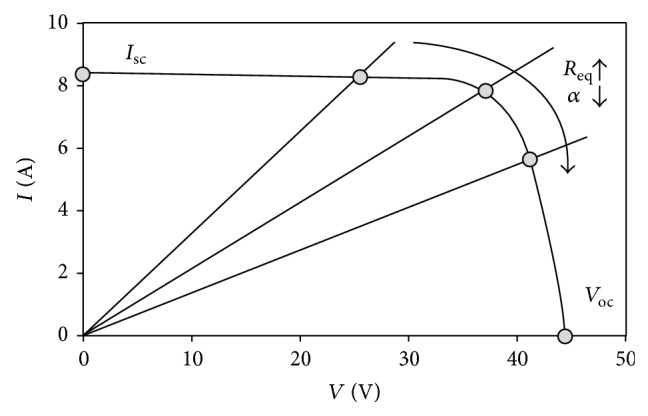
Effect of Boost-converter duty cycle (i.e., the equivalent resistor) variations on the operational point of a solar panel.

Taking the above expression into account, *R*_eq_ increases if *α* decreases which, considering the* I*-*V* curve of a solar panel, entails an increase of the voltage output from the aforementioned panel (see Figure 11). Therefore, the task of an MPPT method would be to find the particular value of the duty cycle which maximizes the output power from the solar panel. As previously mentioned, in the following subsections four MPPT methods are analyzed using the proposed solar panel method, the corresponding algorithms being adapted from the work by Dolara [31].

### 4.1. Constant Voltage Method

This is the simplest MPPT method among the four considered methods. It is based on maintaining the operating voltage of the panel, *V*, as close as possible to a reference value, *V*_ref_. This reference value can be based on the data supplied by the manufacturer. Although this method represents an improvement in terms of energy efficiency when compared to the case of no MPPT method, it is also true that it can be only optimized for one ambient condition. Therefore, the operational point will not be coincident with the maximum power point of the* I*-*V* curve for other sun irradiance and temperature conditions.

**Figure 12:**
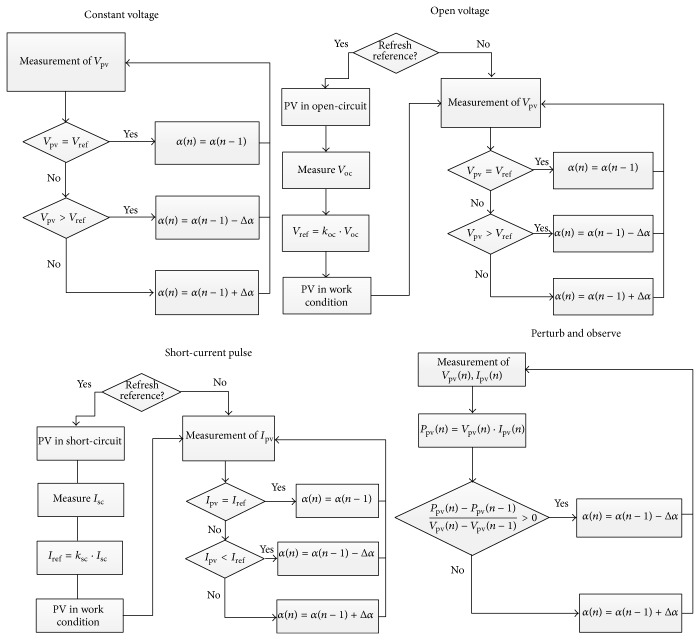
Block-diagrams of the different MPPT used in the present work.

As can be observed in the corresponding block-diagram of Figure 12, if solar panel output voltage, *V*, is higher than the reference value, *V*_ref_, then the duty cycle, *α*, should be reduced, as indicated in the Boost-converter analysis. Otherwise, if *V* < *V*_ref_ it will have to be increased to obtain the opposite effect.

### 4.2. Open Voltage Method

This method is based on the principle that the MPP voltage is always a constant fraction of the open-circuit voltage (i.e., *V*_mp_/*V*_oc_ = *k*_oc_), no matter under which sun irradiance and temperature levels the solar panel is operating. This consideration closely matches reality, although the value of the relationship depends on the solar panel being studied and, as previously suggested, is not entirely constant in the face of sun irradiance and temperature variations. A major drawback of this method is the need to know the open-circuit voltage for given conditions. This voltage level is normally measured by opening the circuit. Logically, this operation stops the flow of energy. Therefore, the number of measurements per unit of time should be adjusted to reduce the loss of energy, bearing in mind that too many measurements will capture variations of the ambient conditions but reduce the solar panel output power. The control principle of the Boost-converter is the same as the previously explained method. The duty cycle, *α*, should be increased so as to lower the panel output voltage and raised to increase it (see the corresponding flow chart in Figure 12).

### 4.3. Short-Current Pulse Method

This method is based on a similar principle to the previous one. In this case the current at MPP is considered to be a fraction of the short-circuit current (i.e., *I*_mp_/*I*_sc_ = *k*_sc_). This fraction is supposed to remain constant in every ambient condition. As with the previous case, this closely matches reality, although there are slight variations depending on sun irradiance and temperature changes. In this case part of the power is lost during the short-circuit current measurements. In this case an increase of the duty cycle, *α*, raises the solar panel output current, the decrease of the duty cycle produces the opposite effect (see the corresponding flow chart in Figure 12).

### 4.4. Perturb and Observe Method

This algorithm is based on continuous modifications of the solar panel operational voltage, checking after each perturbation if the generated power has increased or decreased. If the power increases the next voltage perturbation will go in the same direction and is changed if the power decreases. This process is indicated in the corresponding block-diagram in Figure 12.

## 5. Results

Simulations were carried out with MATLAB. The solar panels were modeled following indications from Section 2. The ambient conditions considered are those described in Section 3, the temperature of the solar panels being calculated with (16). In all cases a 10 ms time step has been considered for data acquisition and control, for every MPPT method analyzed. Regarding the short-current pulse method and the open voltage method a measurement of *I*_sc_ and *V*_oc_ was taken every 3 seconds (300 time steps). Duty cycle was varied in steps of Δ*α* = 0.005 for all methods, the initial value in every calculation being *α*_0_ = 0.12. The control parameters corresponding to the studied methods were optimized, for the solar panel considered in the present work, following these criteria.

(i) Constant voltage method: for the reference voltage value, *V*_ref_, the voltage level at MPP, *V*_mp_, indicated by the manufacturer at 35°C, was selected:
  (21) $$\begin{array}{c} V_{\text{ref}}=31.07-0.41\cdot 10=27.2\;\text{V}. \end{array}$$
(ii) Open voltage method: as control constant, *k*_oc_, the ratio of voltage level at MPP, *V*_mp_, to the open-circuit voltage, *V*_oc_, at STC, was chosen:
  (22) $$\begin{array}{c} k_{\text{oc}}=\frac{31.3}{39.1}=0.8. \end{array}$$
(iii) Short-current pulse method: as control constant, *k*_sc_, the ratio of current level at MPP, *I*_mp_, to the short-circuit current, *I*_sc_, at STC, was chosen:
  (23) $$\begin{array}{c} k_{\text{sc}}=\frac{8.96}{9.5}=0.94. \end{array}$$

**Figure 13:**
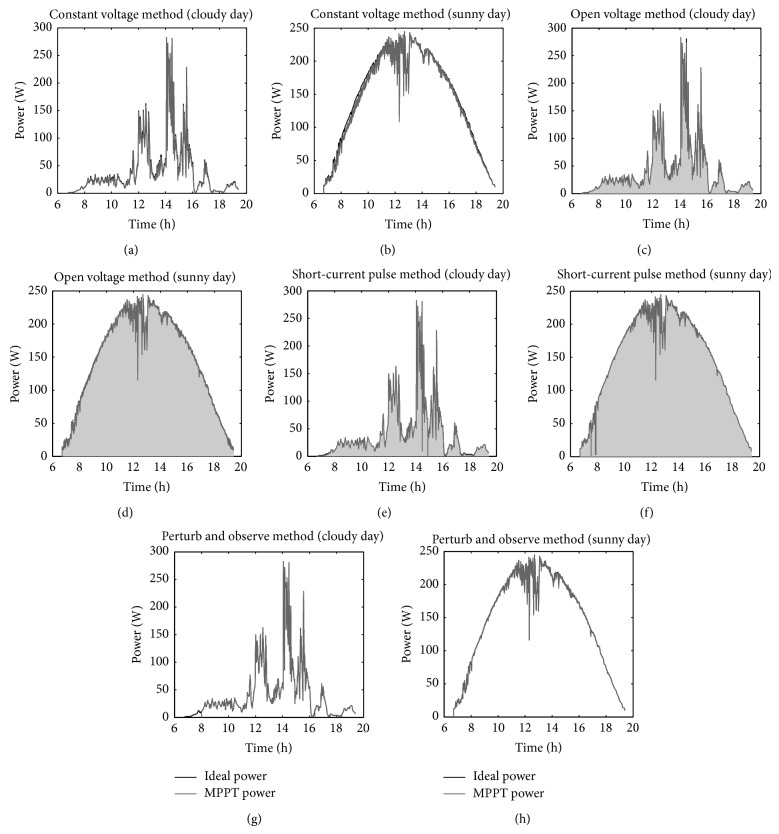
Power produced by YL280C-30b monocrystalline solar panel calculated with the studied MPPT methods, for ambient conditions measured at the Goddard Space Flight Center (GSFC) on May 13, 1971 (cloudy day), and May 14, 1971 (sunny day). Maximum extractable power (ideal) has been also included.

Results are included in Table 2 and Figure 13, together with the maximum extractable power from the solar panel obtained with expressions (13) and (14). The high efficiency obtained for all MPPT methods can be explained as in every case an ideal DC-DC converter (without energy losses) has been considered. The best performance of MPPT method (for the studied conditions) seems to be the perturb and observe method, the reason being the noninterrupted power extraction from the solar panel. The worst performance of MPPT method is the constant voltage method, as it shows the larger influence from the ambient conditions. The open voltage and short-current pulse methods show a quite good performance, the grey zone below the plots being produced by the switching that disconnect the solar panel to perform the measurements of *V*_oc_ and *I*_sc_. The better efficiency showed by the short-current pulse method when compared to the open voltage method could be explained as the ratio *I*_mp_/*I*_sc_ is less dependent on the temperature than the ratio *V*_mp_/*V*_oc_.

Logically, the efficiency of the studied MPPT methods is worse in case of cloudy days than in case of sunny days. This is clear due to the faster variations on the ambient conditions.

## 6. Case Study

**Figure 14:**
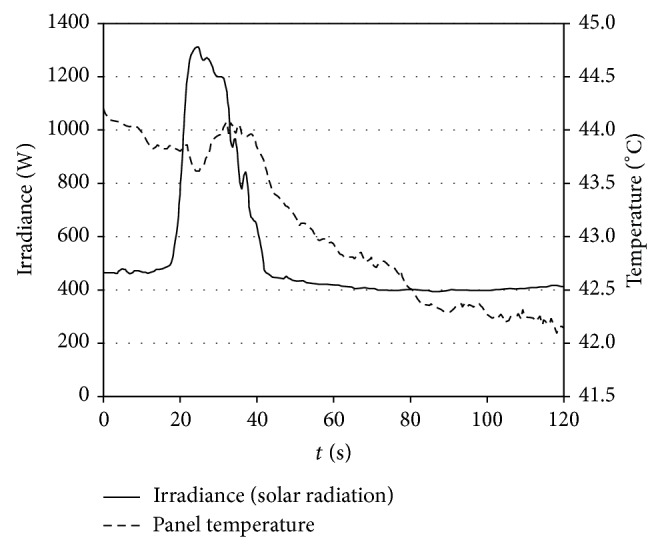
Ambient conditions (irradiance and solar panels temperature) during operation of the photovoltaic facility studied from [50].

In order to check the proposed methodology, a real photovoltaic facility, whose behavior was measured, together with the ambient conditions, by Houssamo et al. [50], has been studied. This photovoltaic facility is formed by eight Q125PI solar panels manufactured by Conergy, organized in four two-panel series connected in parallel. The characteristics of the panels are included in Table 3, whereas the sun radiation and the panel temperature during the measurements are included in Figure 14.

**Figure 15:**
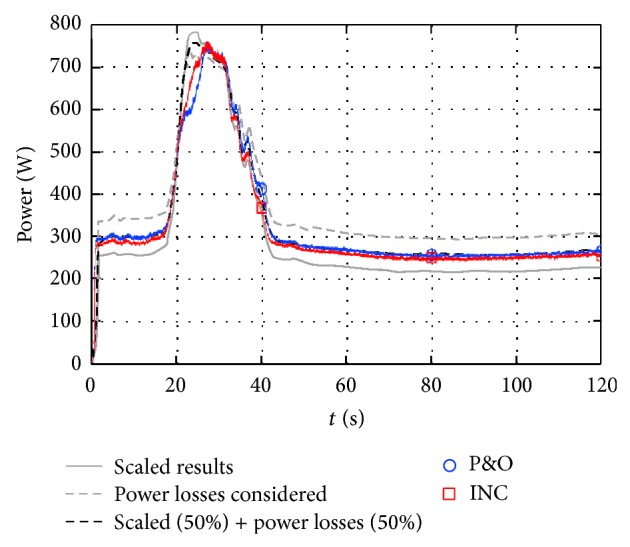
Power from an 8-panel photovoltaic facility calculated with the proposed methodology and P&O MPPT algorithm (scaled results and results with power losses considered). The results from the testing measurements [50] (considering P&O and INC MPPT algorithms) are also included in the figure (P&O: blue line; INC: red line).

The procedure described in Section 4 was followed in order to model the studied photovoltaic facility. The extracted power was calculated using the perturb and observe (P&O) MPPT algorithm. This power was compared to the measured one from the facility [50], which was programmed following two different MPPT algorithms, perturb and observe (P&O) and incremental conductance (INC). The results from the simulation show a higher extracted power when compared to the behavior measured directly on the facility, this discrepancy between results being explained as no power losses (wiring, connections, dirt over the panels, degradation, losses at the Boost-converter, etc.) were taken into account in the simulation carried out. Bearing in mind that no information regarding these losses was included in [50], the results were scaled down by multiplying by a constant (0.64) and, as a result, a much better correlation was obtained (see Figure 15). Also, an equivalent resistor can be then considered in order to take into account the power losses. The results corresponding to the simulation carried out with a 1.375 Ω resistor connected in series with each pair of solar panels are included in Figure 15. A quite good correlation between the results obtained with the present methodology and the ones measured by Houssamo et al. [50] can be observed in the figure. To obtain a better approximation to the measured results, a combination of the scaled results (losses proportional to the extracted power like the ones produced by dirt in the panels) and the losses resulting from electrical current (taken into account with equivalent resistors) should be considered. This result has been included in Figure 15 combining both kinds of power losses at 50% each one. An excellent correlation with the measured extracted power can be observed, the higher deviation being located where the effect of the MPPT algorithm selection is relevant. The highest differences in extracted power between both MPPT algorithms selected by the authors of [50] were measured in period from 20 s to 25 s, which is precisely the period where the higher deviation of the results obtained by present methodology from the measurement results is observed.

## 7. Conclusions

In the present work a solar panel modelling to analyze MPPT methods is described. The most significant conclusions derived from the work are the following.

- It represents a simple and quick-calculation methodology to obtain a solar panel model to perform MPPT simulations, from the manufacturers' datasheet.
- The developed method takes into account for the calculations possible variations on the ambient conditions (sun irradiation and solar cells temperature).
- The proposed analytical methodology allows fast MPPT methods comparison.
- Based on the developed methodology the performance of a specific MPPT method can be foreseen when applied to a particular solar panel from which only manufacturer's datasheet information is available.
- Simple, accurate, and low calculation resources demanding way to obtain the maximum extractable power from solar panels are presented.
- The feasibility of the described methodology has been checked with four different MPPT methods applied to a commercial solar panel, within a day, and under realistic ambient conditions. Also a case study was analyzed, the results being compared to testing measured with good correlation.
- Finally, it should also be mentioned that although the parameter-extraction analytical model which is the core of the proposed methodology has proven to be as accurate as several numerical methods [35], the most recently developed algorithms can be an improvement in terms of accuracy (at least if the photovoltaic device is partially shaded).

## Figures and Tables

**Table 1:** Characteristic points of YL280C-30b solar panel (Yingli Solar), included in the manufacturer's datasheets [ref] at Standard Test Conditions (STC): 1000 W/m^2^ irradiance, 25°C cell temperature, and AM1.5 g spectrum according to EN 60904-3 [17].

YL280C-30b (monocrystalline)			
*n*	60	*T*_*r*_ (°C)	25
*P*_mp_ (W)	280	*γ* (%/°C)	−0.42
*I*_mp_ (A)	8.96	*αI*_mp_ (%/°C)	—
*V*_mp_ (V)	31.3	*βV*_mp_ (%/°C)	−0.41
*I*_sc_ (A)	9.50	*αI*_sc_ (%/°C)	0.04
*V*_oc_ (V)	39.1	*βV*_oc_ (mV/°C)	−0.31

**Table 2:** Energy obtained from YL280C-30b monocrystalline solar panel calculated with ambient conditions measured at the Goddard Space Flight Center (GSFC) on May 13, 1971 (cloudy day), and May 14, 1971 (sunny day).

MPPT method	Sunny day			Cloudy day		
	Energy [W h]	*η*_MPPT_	Rank	Energy [W h]	*η*_MPPT_	Rank
Ideal	1880	1	—	526	1	—
Constant voltage	1826	0.971	4	496	0.943	4
Open voltage	1860	0.989	3	513	0.976	3
Short-current pulse	1867	0.993	2	518	0.985	2
Perturb and observe	1871	0.995	1	522	0.992	1

**Table 3:** Characteristic points of Q125PI solar panel (Conergy), included in the manufacturer's datasheets at Standard Test Conditions (STC): 1000 W/m^2^ irradiance, 25°C cell temperature, and AM1.5 g spectrum according to EN 60904-3 [17].

Conergy Q125Pl (polycrystalline)			
*n*	36	*T*_*r*_ (°C)	25
*P*_mp_ (W)	125	*γ* (%/°C)	−0.426
*I*_mp_ (A)	7.36	*αI*_mp_ (%/°C)	—
*V*_mp_ (V)	17	*βV*_mp_ (%/°C)	−0.352
*I*_sc_ (A)	7.94	*αI*_sc_ (%/°C)	0.035
*V*_oc_ (V)	21	*βV*_oc_ (V/°C)	−0.074

## References

[1] Pindado S. (2006). *Elementos de Transporte Aéreo*.

[2] Aminzadeh F., Pindado S. How has Spain become a leader in the wind energy industry during the last decade? (An analysis of influential factors on the development of wind energy in Spain).

[3] Vázquez C. T. (2008). *Energía Solar Fotovoltaica*.

[4] Hernandez V. R. (2010). *Solar Thermal Power. History of a Research Success*.

[5] Dinçer F. (2011). The analysis on photovoltaic electricity generation status, potential and policies of the leading countries in solar energy. *Renewable and Sustainable Energy Reviews*.

[6] Solangi K. H., Islam M. R., Saidur R., Rahim N. A., Fayaz H. (2011). A review on global solar energy policy. *Renewable and Sustainable Energy Reviews*.

[7] Singh G. K. (2013). Solar power generation by PV (photovoltaic) technology: a review. *Energy*.

[8] Karam N. H., King R. R., Terence Cavicchi B. (1999). Development and characterization of high-efficiency Ga_0.5_In_0.5_P/GaAs/Ge dual- and triple-junction solar cells. *IEEE Transactions on Electron Devices*.

[9] Miles R. W. (2006). Photovoltaic solar cells: choice of materials and production methods. *Vacuum*.

[10] Takamoto T., Agui T., Yoshida A. World's highest efficiency triple-junction solar cells fabricated by inverted layers transfer process.

[11] Green M. A., Emery K., Hishikawa Y., Warta W., Dunlop E. D. (2012). Solar cell efficiency tables (version 39). *Progress in Photovoltaics: Research and Applications*.

[12] Moliton A., Nunzi J.-M. (2006). How to model the behaviour of organic photovoltaic cells. *Polymer International*.

[13] Bendib T., Djeffal F., Arar D., Meguellati M. Fuzzy-logic-based approach for organic solar cell parameters extraction.

[14] Zuo L., Yao J., Li H., Chen H. (2014). Assessing the origin of the S-shaped I-V curve in organic solar cells: an improved equivalent circuit model. *Solar Energy Materials and Solar Cells*.

[15] Esram T., Chapman P. L. (2007). Comparison of photovoltaic array maximum power point tracking techniques. *IEEE Transactions on Energy Conversion*.

[16] Reza Reisi A., Hassan Moradi M., Jamasb S. (2013). Classification and comparison of maximum power point tracking techniques for photovoltaic system: a review. *Renewable and Sustainable Energy Reviews*.

[17] Yingli Energy Panda 60 Cell 40 mm Series.

[18] Salas V., Olías E., Barrado A., Lázaro A. (2006). Review of the maximum power point tracking algorithms for stand-alone photovoltaic systems. *Solar Energy Materials and Solar Cells*.

[19] Bhatnagar P., Nema R. K. (2013). Maximum power point tracking control techniques: State-of-the-art in photovoltaic applications. *Renewable and Sustainable Energy Reviews*.

[20] Goudar M. D., Patil B. P., Kumar V. (2010). A review of improved maximum peak power tracking algorithms for photovoltaic systems. *International Journal of Electrical Engineering*.

[21] Ali A. N. A., Saied M. H., Mostafa M. Z., Abdel- Moneim T. M. A survey of maximum PPT techniques of PV systems.

[22] Ishaque K., Salam Z. (2013). A review of maximum power point tracking techniques of PV system for uniform insolation and partial shading condition. *Renewable and Sustainable Energy Reviews*.

[23] Hua C., Shen C. Comparative study of peak power tracking techniques for solar storage system.

[24] Hohm D. P., Ropp M. E. (2003). Comparative study of maximum power point tracking algorithms. *Progress in Photovoltaics: Research and Applications*.

[25] Faranda R., Leva S., Milano P., Leonardo P. (2008). Energy comparison of MPPT techniques for PV systems department of energy. *Wseas Transactions on Power Systems*.

[26] Faranda R., Leva S., Maugeri V. MPPT techniques for PV systems: energetic and cost comparison.

[27] Berrera M., Dolara A., Faranda R., Leva S. Experimental test of seven widely-adopted MPPT algorithms.

[28] Karanjkar D. S., Chatterji S., Shimi S. L., Kumar A. Real time simulation and analysis of maximum power point tracking (MPPT) techniques for solar photo-voltaic system.

[29] Choi K. K., Llorens J.-C., Maral G., Barnaba A. Application of the maximum power point tracking method to the adaptative power supply sub-system of a microsatellite.

[30] de Manuel C., Cubas J., Pindado S. On the simulation of the UPMSat-2 microsatellite power.

[31] Dolara A. (2009). Energy comparison of seven MPPT techniques for PV systems. *Journal of Electromagnetic Analysis and Applications*.

[32] Esram T., Kimball J. W., Krein P. T., Chapman P. L., Midya P. (2006). Dynamic maximum power point tracking of photovoltaic arrays using ripple correlation control. *IEEE Transactions on Power Electronics*.

[33] D'Souza N. S., Lopes L. A. C., Liu X. (2010). Comparative study of variable size perturbation and observation maximum power point trackers for PV systems. *Electric Power Systems Research*.

[34] Ding K., Bian X., Liu H., Peng T. (2012). A MATLAB-simulink-based PV module model and its application under conditions of nonuniform irradiance. *IEEE Transactions on Energy Conversion*.

[35] Cubas J., Pindado S., Victoria M. (2014). On the analytical approach for modeling photovoltaic systems behavior. *Journal of Power Sources*.

[36] Cubas J., Pindado S., de Manuel C. (2014). Explicit expressions for solar panel equivalent circuit parameters based on analytical formulation and the Lambert W-function. *Energies*.

[37] Cubas J., Pindado S., Farrahi A. New method for analytical photovoltaic parameter extraction.

[38] Jain A., Kapoor A. (2005). A new approach to study organic solar cell using Lambert W-function. *Solar Energy Materials and Solar Cells*.

[39] Villalva M. G., Gazoli J. R., Ruppert Filho E. Modeling and circuit-based simulation of photovoltaic arrays.

[40] Chan D. S. H., Phang J. C. H. (1987). Analytical methods for the extraction of solar-cell single- and double-diode model parameters from I-V characteristics. *IEEE Transactions on Electron Devices*.

[41] Phang J. C. H., Chan D. S. H., Phillips J. R. (1984). Accurate analytical method for the extraction of solar cell model parameters. *Electronics Letters*.

[42] Villalva M. G., Gazoli J. R., Filho E. R. (2009). Comprehensive approach to modeling and simulation of photovoltaic arrays. *IEEE Transactions on Power Electronics*.

[43] Cuce P. M., Cuce E. (2012). A novel model of photovoltaic modules for parameter estimation and thermodynamic assessment. *International Journal of Low-Carbon Technologies*.

[44] Rauschenbach H. S. (1980). *Solar Cell Array Design Handbook: The Principles and Technology of Photovoltaic Energy Conversion*.

[45] Hussein K. H., Muta I., Hoshino T., Osakada M. (1995). Maximum photovoltaic power tracking: an algorithm for rapidly changing atmospheric conditions. *IEE Proceedings: Generation, Transmission and Distribution*.

[46] Hua C., Shen C. Comparative study of peak power tracking techniques for solar storage system.

[47] Thekaekara M. P. (1976). Solar radiation measurement: techniques and instrumentation. *Solar Energy*.

[48] National Oceanic and Atmospheric Administration’s *National Weather Service*.

[49] Skoplaki E., Palyvos J. A. (2009). Operating temperature of photovoltaic modules: a survey of pertinent correlations. *Renewable Energy*.

[50] Houssamo I., Locment F., Sechilariu M. (2010). Maximum power tracking for photovoltaic power system: development and experimental comparison of two algorithms. *Renewable Energy*.

